# Stability of the Acetic Acid-Induced Bladder Irritation Model in Alpha Chloralose-Anesthetized Female Cats

**DOI:** 10.1371/journal.pone.0073771

**Published:** 2013-09-09

**Authors:** F. Aura Kullmann, Grace I. Wells, Christopher L. Langdale, Jihong Zheng, Karl B. Thor

**Affiliations:** Urogenix Inc./Astellas, Durham, North Carolina, United States of America; University of Louisville, United States of America

## Abstract

Time- and vehicle-related variability of bladder and urethral rhabdosphincter (URS) activity as well as cardiorespiratory and blood chemistry values were examined in the acetic acid-induced bladder irritation model in α-chloralose-anesthetized female cats. Additionally, bladder and urethra were evaluated histologically using Mason trichrome and toluidine blue staining. Urodynamic, cardiovascular and respiratory parameters were collected during intravesical saline infusion followed by acetic acid (0.5%) to irritate the bladder. One hour after starting acetic acid infusion, a protocol consisting of a cystometrogram, continuous infusion-induced rhythmic voiding contractions, and a 5 min “quiet period” (bladder emptied without infusion) was precisely repeated every 30 minutes. Administration of vehicle (saline i.v.) occurred 15 minutes after starting each of the first 7 cystometrograms and duloxetine (1mg/kg i.v.) after the 8^th^. Acetic acid infusion into the bladder increased URS-EMG activity, bladder contraction frequency, and decreased contraction amplitude and capacity, compared to saline. Bladder activity and URS activity stabilized within 1 and 2 hours, respectively. Duloxetine administration significantly decreased bladder contraction frequency and increased URS-EMG activity to levels similar to previous reports. Cardiorespiratory parameters and blood gas levels remained consistent throughout the experiment. The epithelium of the bladder and urethra were greatly damaged and edema and infiltration of neutrophils in the lamina propria of urethra were observed. These data provide an ample evaluation of the health of the animals, stability of voiding function and appropriateness of the model for testing drugs designed to evaluate lower urinary tract as well as cardiovascular and respiratory systems function.

## Introduction

Bladder and urethra store and periodically release urine in a coordinated manner. Voiding is achieved via release of acetylcholine/adenosine triphosphate (ACh/ATP) and nitric oxide (NO) from the parasympathetic fibers to concomitantly contract the bladder and relax the urethra, respectively [1]. Continence during the storage phase is achieved via tonic release of norepinephrine (NE) to relax the bladder and contract the urethra. In addition, the urethral rhabdosphincter (URS) maintains continence especially when stress is applied to the bladder [2,3].

The cat is a commonly used animal model of lower urinary tract (LUT) function because of its similarity to humans in a number of characteristics, including anatomy, innervation and function. One important similarity to humans and different from rodents is that during storage the URS-EMG activity is elevated, while during voiding it is strongly inhibited to allow urine release [2,3]. Behavioral, urodynamic, and/or electrophysiological techniques have been used for understanding LUT physiology and pharmacology in conscious [4–8], alpha chloralose-anesthetized [9–19], normal adult [9,17,20–23] and neonatal cats [6,24]. Models of overactive bladder (OAB) [8,11,25,26], stress urinary incontinence (SUI) [10,27], spinal cord injury [7,28–30], and interstitial cystitis [18,31] have also been developed and/or studied in the cat. Many acute OAB [8,11,25,26] and SUI [10,27] models frequently employ bladder irritation, commonly using dilute acetic acid (0.25-0.5%). Although this acetic acid-induced bladder irritation model in alpha chloralose-anesthetized female cats has been used for many years in various pharmacological and physiological studies of LUT function, the stability across time and multiple vehicle administrations (i.e. across a 5-8 hour period required for multiple drug administrations to create cumulative dose–response characteristics and antagonist administration) has not been published. In this study we evaluate the stability of bladder and rhabdosphincter EMG activity during a 6-7 hour experiment with 7 intravenous saline vehicle injections to mimic a dose–response protocol. We include an intravenous administration of duloxetine, a combined serotonin and norepinephrine reuptake inhibitor, at the end of the current standardized protocol to compare its effects with previous studies [10].

Another important area with a paucity of data includes the effects of prolonged anesthesia on cardiovascular, respiratory and blood acid–base regulation in chloralose-anesthetized animals. alpha-chloralose is a widely used anesthetic for LUT, cardiorespiratory [32–35], and sensory function studies because it preserves most reflexes compared to other anesthetics (e.g. isoflurane, pentobarbital [36–38], although it can alter them [39,40]. Only a few studies in dog [40–42], and none in cat to our knowledge, have addressed the effects of long-term chloralose anesthesia on homeostatic physiology. Given the importance of stable experimental conditions and the paucity of data, we therefore also characterized cardiorespiratory and blood chemistry variability throughout the experiment.

Finally, the extent of bladder and urethra damage induced by prolonged infusion of acetic acid into the bladder in the cat has not been reported. Here we used histological methods to qualitatively assess the extent of tissue damage during a 6-7 hour experiment.

Together, these parameters provide an extensive evaluation of the health of the animals, stability of voiding function and appropriateness of the model for testing drugs designed for SUI, OAB, and possibly cardiovascular and respiratory diseases.

## Methods

### Animals

Studies were conducted in 6-8 month old (2.1 to 3.2 kg; n=16) female domestic short hair cats obtained from Liberty Research Inc. (Waverly, NY).

### Ethics statement

All protocols were approved by Urogenix Institutional Animal Care and Use Committee which adheres to NIH Guidelines for the Care and Use of Laboratory Animals. Urogenix is an Association for Assessment and Accreditation of Laboratory Animal Care (AAALAC) accredited facility.

### Surgical procedures

Cats were initially anesthetized with isoflurane (2%) and two i.v. catheters (SurFlash; Terumo, Somerset, NJ) were placed in each of the upper cephalic veins for anesthesia and drug delivery. The carotid artery was cannulated using a PE-50 tubing connected to an ArgoTrans pressure transducer (Argon Medical Devices, Athens, TX) for measuring blood pressure. A trachea tube consisting of a Murphy endotracheal tube (Teleflex Medical, Bannockburn, IL) was placed to allow measurement of expired CO_2_ using an AD Instrument Gas Analyzer (Colorado Springs, CO) and to monitor respiration rate. The urinary bladder was cannulated through the dome using a 14G AngioCath (Becton Dickinson, Sandy, UT) attached to PE 90 tubing. This was connected to an infusion pump for bladder infusion as well to an ArgoTrans pressure transducer for bladder pressure measurements. Electrodes (PTFE insulated 10% platinum/iridium 10IR5T wire Sigmund Cohn Corp, Mount Vernon, NY) for measuring urethral rhabdosphincter electromyogram (URS-EMG) activity were placed into the rhabdosphincter through the vaginal opening, and their position was verified after a necropsy at the end of the experiment. A reference electrode was placed in the subcutaneous tissue. URS-EMG activity was sampled at a rate of 1 kHz and recorded using a Grass amplifier (P511AC; Astro-Med, West Warwick, RI). Over the course of 1h, while performing surgical procedures described above, isoflurane concentrations were gradually reduced (0.1-0.2% decremented steps every 3-6 minutes) with subsequent administration of small doses of intravenous chloralose (5-10 mg/kg incremental steps every 3-6 minutes) to maintain anesthesia at a surgical level, up to a final dose of 65-75 mg/kg, at which time isoflurane was no longer required. At this time and throughout the experiment 5 mg/kg/h i.v. alpha-chloralose was delivered via an infusion pump to maintain a stable level of anesthesia. This continuous infusion of alpha-chloralose was used because in pilot studies boluses (10-12mg/kg; n=3 cats) given while the cats were under alpha-chloralose anesthesia, greatly inhibited URS-EMG activity while it had less impact on bladder activity (Figure S1).

Voiding contractions were elicited by intravesical infusion of warm saline (0.9% NaCl Baxter, Deerfield, IL; 28-32^o^C). To create an acute OAB model, the bladder was irritated using dilute acetic acid solution (saline with 0.5% glacial acetic acid, Fisher Scientific, Fair Lawn, NJ). During the surgery and throughout the experiment the animals were placed on heating pads and body temperature was monitored with an esophageal thermometer and maintained at 38-39^o^C. At the end of the experiment, the animals were sacrificed by an overdose of alpha-chloralose anesthetic, followed by 4M KCl.

### Experimental protocol (figure 1)

**Figure 1:**
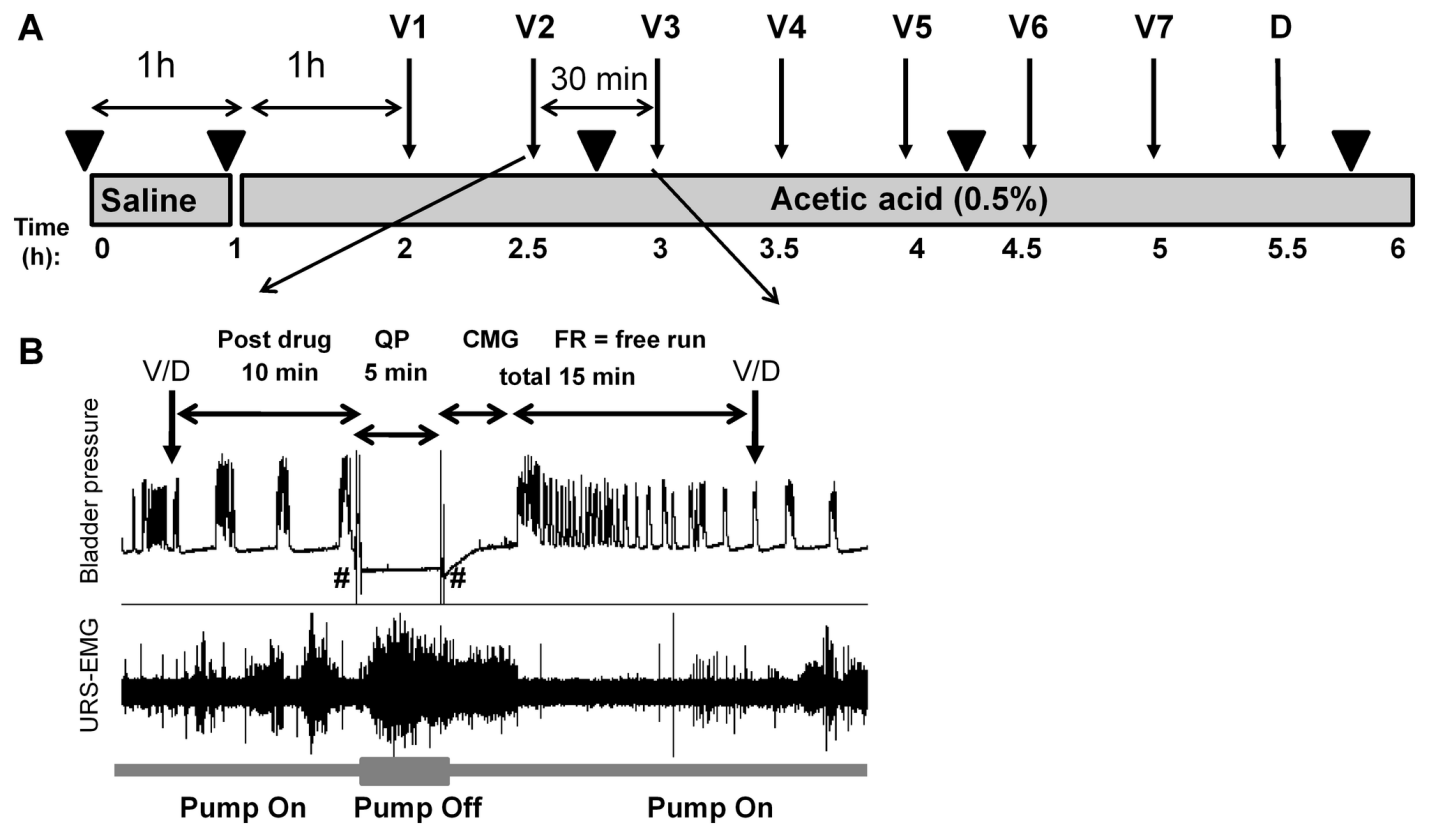
Experimental protocol. A. Schematic of the protocol design. The bladder was infused with saline for 1h, followed by saline with 0.5% acetic acid for the remaining of the experiment. V1-V7 indicate vehicle (saline 0.5mg/kg) given i.v. every 30 minutes. D indicates duloxetine, 1mg/kg i.v. Numbers under grey boxes show time in hours from the beginning of the recordings. B. Inset illustrates the 30 minute four-step protocol, which is repeated throughout the experiment following each vehicle or drug (V/D) delivery. The steps are: post drug (PD), quiet period (QP), cystometrogram (CMG) and free run cystometry (FR). PD is a period of 10 minutes of continuous cystometry immediately following drug injection. After this period, the infusion pump is stopped and the bladder is drained and allowed to rest for 5 minutes, which defines the QP. At the end of the QP the bladder is drained again, the infusion pump turned on and a CMG is started. This period is followed by continuous cystometry (FR). CMG and FR periods total 15 minutes. # indicates bladder emptying. ▼ indicates time points for drawing blood samples (0.5ml) for blood gas analysis.

The bladder was initially infused for 1h with saline at 0.9 to 1.8 ml/min, a rate that produced voiding every 5-8 minutes. The infusion solution was then changed to 0.5% acetic acid in saline (pH ~3-4) for the remainder of the experiment. Dosing commenced 1h after accommodation to acetic acid and consisted of 7 doses of vehicle (0.5 ml/kg saline, i.v.) injected every 30 minutes followed by a single dose of duloxetine (1 mg/kg i.v.). Ten minutes after each injection, the bladder was emptied and the infusion pump was turned off for a 5 minute “quiet period” (QP) period to record URS-EMG activity in the absence of bladder activity. A CMG followed by a free run cystometry period were recorded over the remaining 15 of the 30 minute dosing block (Figure 1). The QP was adopted because acetic acid irritation strongly excites the bladder, reducing bladder capacity, thus the bladder contracts at smaller infused volumes. The reduction in bladder capacity (measured as a reduction of CMG duration), means shorter duration of the period when sphincter activity is evaluated. This may not consistently allow assessments of drug effects and may increase data variability. Thus, for a more reliable assessment of sphincter activity in the absence of inhibition due to bladder contraction, and for a longer, defined and better controlled period of time, the QP was adopted.

### Blood gas analysis

Prior to alpha-chloralose anesthesia, after 1 hour of saline infusion into the bladder, then at 1.5 hour intervals, blood samples (0.5 ml) were collected from the carotid artery into syringes containing 3.2 IU of heparin (GasLyte Totowa, NJ). Samples were analyzed for pH, oxygen/hemoglobin status, and electrolyte content, using an IDEXX Vetstat analyzer (Westbrook, ME). The values measured prior to the start of alpha-chloralose anesthesia were not included in data analysis because the cat was under isoflurane anesthesia and supplemented with 100% O_2_ which artificially increased the PO_2_ levels.

### Data analysis

**Figure 2:**
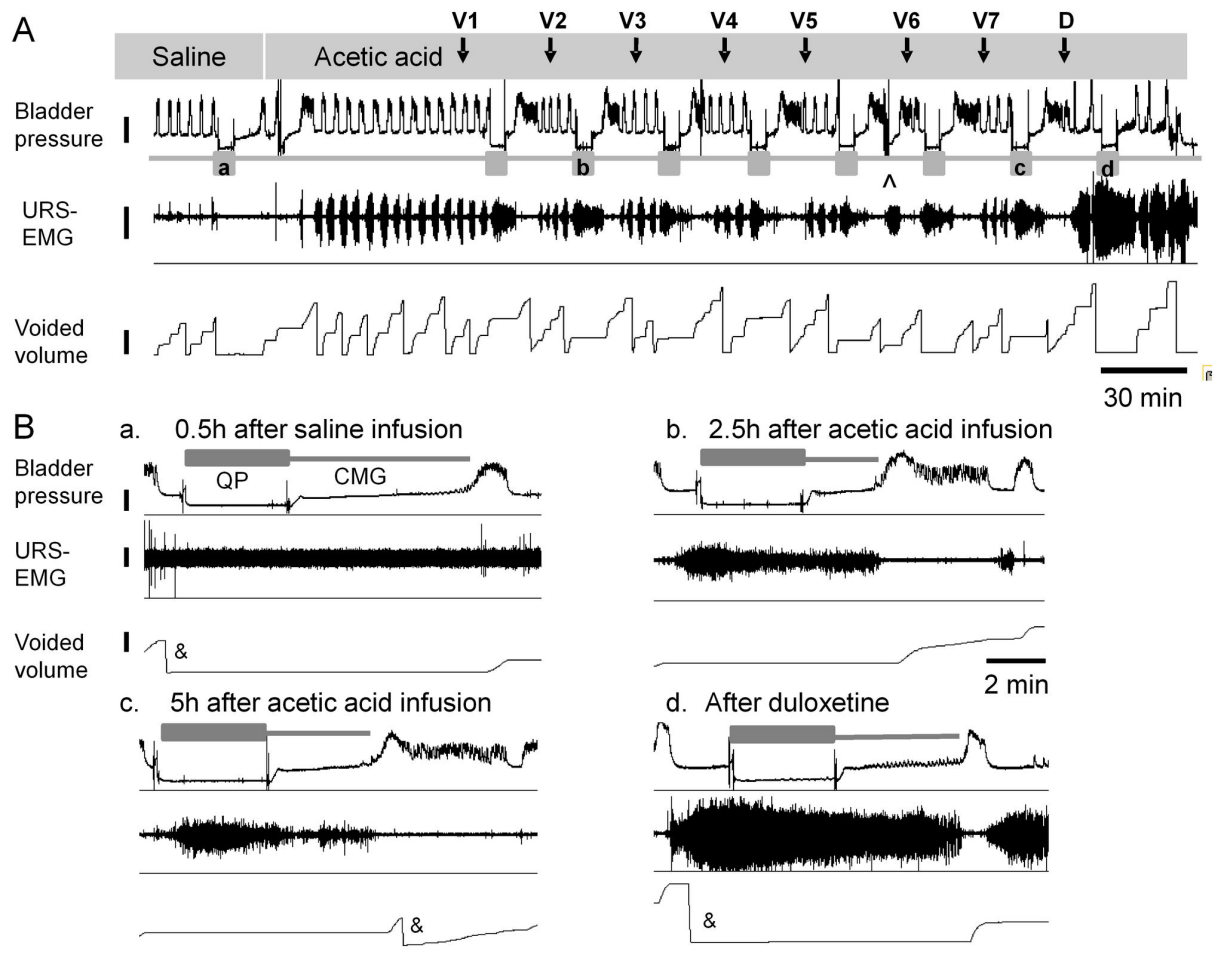
Typical recording traces. A. Bladder pressure, URS-EMG activity and voided volume recorded while bladder was infused with saline followed by acetic acid. Grey boxes underneath bladder pressure trace indicate quiet periods. Letters inside grey boxes correspond to insets enlarged in B. Calibration bars are 10 mmHg for bladder pressure, 1 µV for URS-EMG activity, 2 ml for voided volume and 30 min for time scale. B. Enlargement of traces illustrating bladder and URS-EMG activity during QP and CMG periods at different time points during the experiment: a) in saline after 0.5 h; b, c) after 2.5 and 5h of acetic acid irritation, respectively; d) after duloxetine 1mg/kg. Scales for bladder pressure, voided volume and time are the same for all insets, 10 mmHg, 10 ml, 2 min, respectively, and are illustrated in inset a. The scale for URS-EMG is 0.1 µV and 0.5 µV in insets a, b-d respectively. Vehicle is saline (0.5 ml/kg i.v.). ^ indicates the time point when bladder infusion syringes were replenished. & indicates resetting the balance collecting the volume voided.

Respiration rate, blood pressure, bladder pressure, micturition volume and URS-EMG activity were recorded using LabChart 7 software (version 7; ADInstruments, Australia) and analyzed using Excel (Microsoft, Redmond, WA) and Prism 5 (GraphPad Software, Inc, San Diego, CA). URS-EMG activity was calculated during QP and CMG. The square root of the mean value of the squared signal (RMS) and mean firing rate (spikes/sec) were measured using LabChart built-in functions. The threshold for discriminating a spike was empirically set to twice the baseline voltage. Baseline electrical noise was measured from the URS-EMG electrodes 10-15 min after euthanasia, and it was subtracted from all values. CMG duration was measured from the time infusion into the empty bladder was started until voiding occurred (Figure 2). Bladder capacity was calculated by multiplying the CMG duration by the infusion rate. Voiding frequency (VF) was calculated as number of voids per minute. Bladder contraction amplitude (BCA) was calculated as the maximal contraction amplitude minus baseline intravesical pressure measured immediately after voiding. VF and BCA were analyzed during free run period (Figure 2). Mean arterial pressure (MAP; mmHg), heart rate (HR; beats/min) and respiration rate (RR; number of respiration cycles per minute), were measured during the quiet periods in order to minimize the changes in blood pressure caused by bladder filling and contraction [43] and analyzed using LabChart built-in functions.

### Drugs

Duloxetine HCl, a serotonin and norepinephrine reuptake inhibitor was purchased from Sequoia Research Products, Pangbourne, UK.

### Histology

Two saline infused and two acetic acid infused cat bladders and urethrae were taken 6-7 hours post cystometry experiments and fixed in 4% PFA overnight followed by 30% sucrose. Tissues were blocked in 10 mm blocks. Cryostat sections (12 µm) were cut from bladder and urethral regions (proximal 1, proximal 2, mid urethra, and vaginal blocks) and mounted on slides for histology. Bladder sections were cut longitudinal (neck to dome), and urethral sections were cut in transverse orientation. Adjacent sections were stained using Masson’s trichrome and toluidine blue using routine protocols. Masson’s trichrome stains collagen fibers in blue, muscle, cytoplasm, keratin muscle fibers in red and nuclei in black [44]. Toluidine blue staining can be used to identify neutrophils based on their morphological appearance, presenting cytoplasmic lysosomal granules [45]. Pictures were taken using an Olympus BX-51 microscope equipped with a Retiga-2000R Fast 1394 Color camera (model RET-2000R-F-CLR-12). The acquisition system was controlled by a DELL T7400 computer with Stereo Investigator software (MBF Bioscience, Williston, VT). Final images were processed using Photoshop CS5 (Adobe Systems Incorporated, San Jose, California).

### Statistics

Results are expressed as mean +/- SEM and analyzed using t-test or non-parametric one-way ANOVA (Friedman Test) followed by Dunn’s Multiple Comparison post-test (significance set at p<0.05) using Prism 5.

## Results

### Bladder contraction and urethral rhabdosphincter-EMG activity

**Figure 3:**
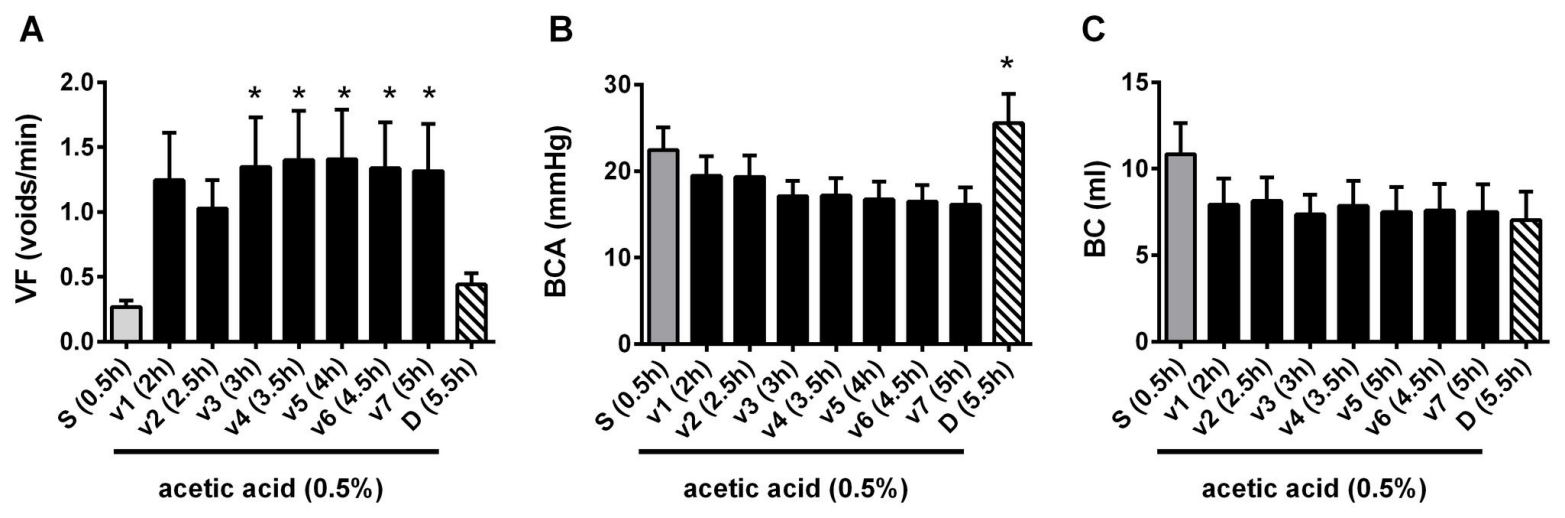
Summary of bladder parameters throughout the experiment. A. Voiding frequency. B. Bladder contraction amplitude. C. Bladder capacity. Data included are from 12 cats. Vehicle is saline (0.5 ml/kg i.v.). Asterisks (*) represent statistically significant differences relative to saline in A and relative to V7 in B, tested with ANOVA followed by Dunn’s posthoc.

**Figure 4:**
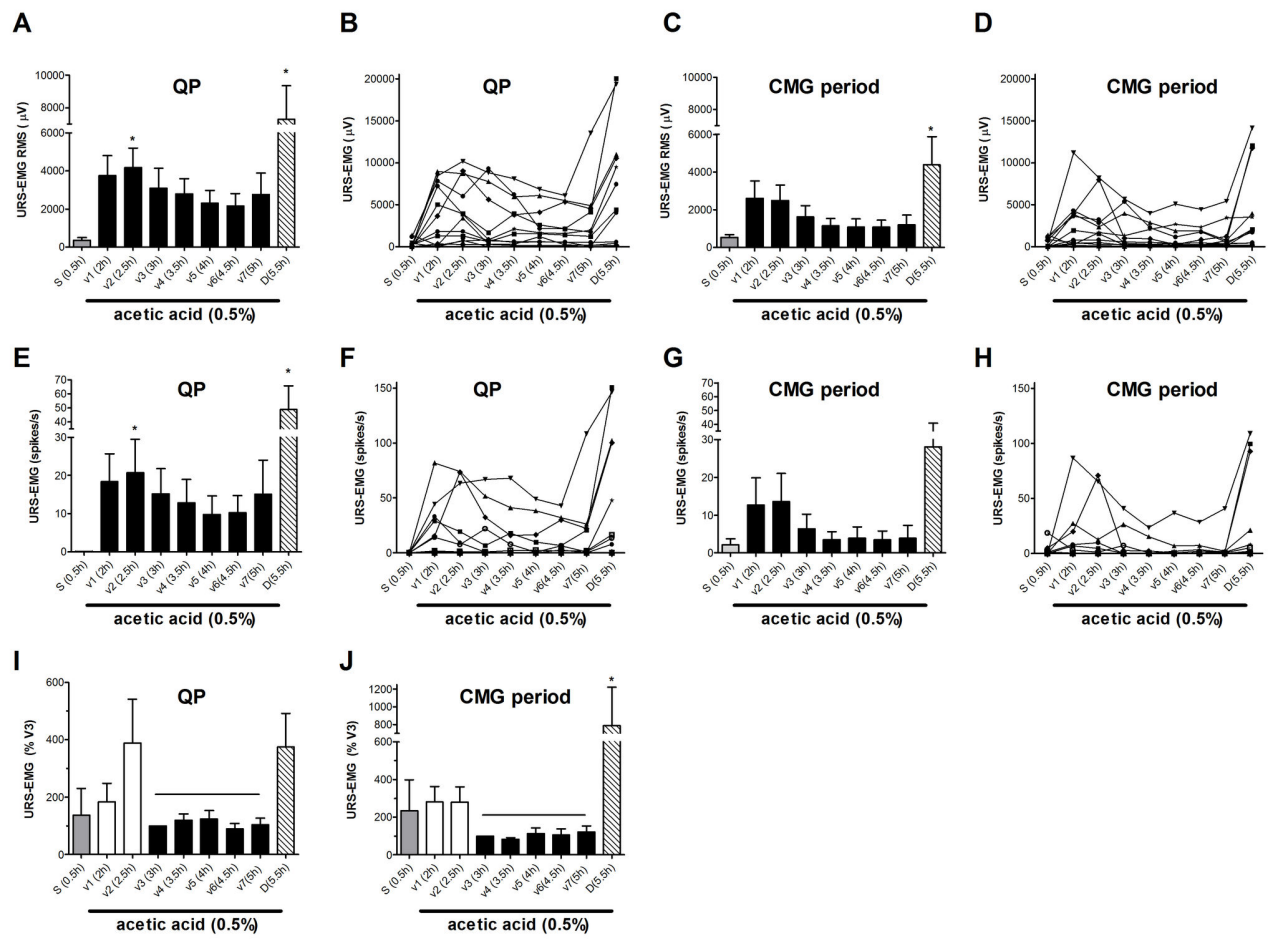
Summary of URS-EMG parameters. A–D. URS-EMG expressed as RMS during QP (A, B) and CMG periods (C, D). A, C show averaged data from 12 cats and B, D show data from individual cats. E–H. URS-EMG expressed as spikes/s during QP (E,F) and CMG periods (G,H). E, G show averaged data from 12 cats and F, H show data from individual cats. I,J. Summary of URS-EMG normalized to V3 to illustrate stability from V3 to V7. Vehicle is saline (0.5 ml/kg i.v.). Asterisks (*) represent statistical significant values relative to S (saline) tested with ANOVA followed by Dunn’s posthoc test.

Saline infusion into the bladder induced rhythmic large amplitude (22.0 ± 2.8 mmHg) contractions associated with voiding (Figure 2). URS-EMG activity was very low. Switching to acetic acid (0.5%, pH ~3-4) infusion for 1h increased voiding frequency, decreased bladder contraction amplitude, and bladder capacity (figures 2, 3). These changes became stable after ~1h and were maintained throughout the duration of the experiment (5-6 hours). Acetic acid also induced a robust but highly variable increase of the URS-EMG activity (both RMS and spikes/s) (Figure 4). In an individual animal, variability in EMG activity remained high across the first 2 vehicle administrations (1.5 hours) and then stabilized across all subsequent vehicle administrations. However, between animals, the magnitude of the increase in EMG activity (RMS and spikes/sec) was high at all time periods (Figure 4 B, D, F, H). Because of this interanimal variability, data were also normalized to URS-EMG activity after vehicle 3 (V3, 2h after acetic acid infusion) (Figure 4 I, J) to capture changes in the lower magnitude EMG increases. Analyses of either normalized or raw data (RMS and spikes/sec) demonstrated that after the 3^rd^ vehicle, URS-EMG activity became relatively stable, varying less than 25% (relative to V3), for the remaining duration of the study (Figure 4 A–H). Throughout the course of the experiment, URS-EMG activity was higher during QP than during CMG (Figure 4), but the increasing and decreasing trends with experimental manipulations were similar. Duloxetine (1 mg/kg) inhibited the acetic acid induced sensitization of most bladder parameters (VF, BCA) a greatly increased URS-EMG activity during both QP and CMG (figures 2-4). Throughout the course of all experiments, reciprocal inhibition of sphincter activity during a bladder contraction and voiding was maintained (Figure 1).

### Vital signs and blood chemistry

**Figure 5:**
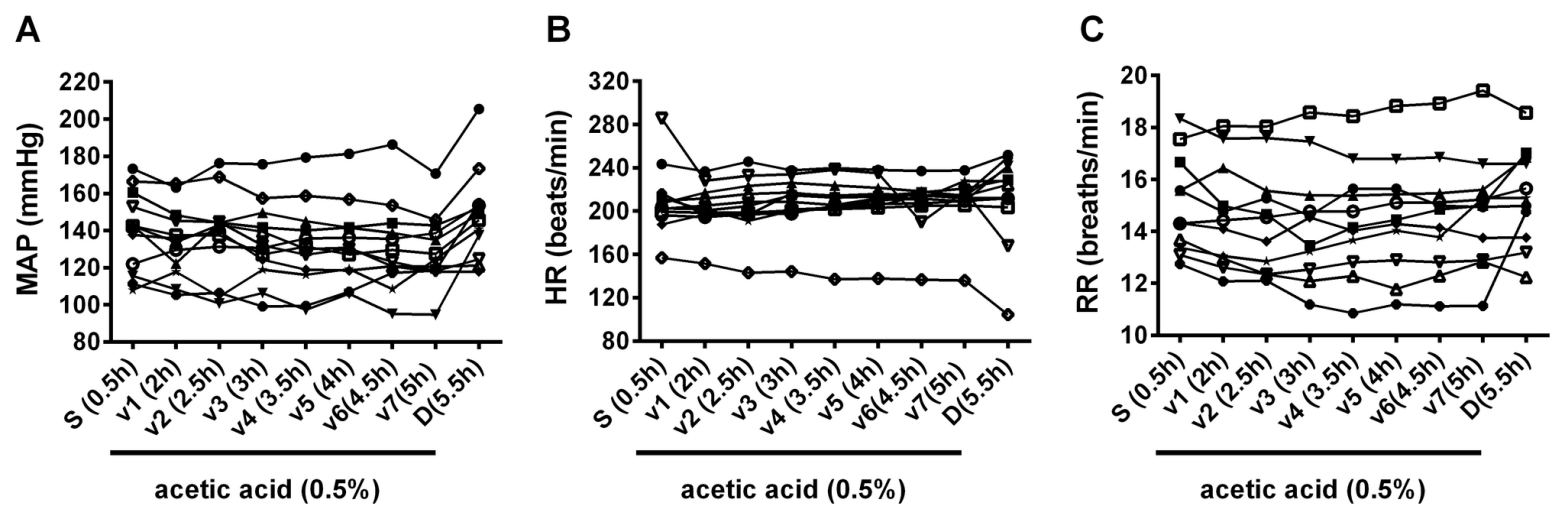
Time course of cardiovascular and respiratory parameters. Raw values from individual cats (each line represents data from an animal) are shown for: . A. Mean arterial pressure (MAP). Data included are from 12 cats. B. Heart rate (HR). Data included are from 12 cats. C. Respiratory rate (RR). Data included are from 11 cats. Averaged values are shown in table 1.

Respiration rate, mean arterial pressure, heart rate, and blood chemistry remained consistent throughout the experiment (table 1, Figure 5). Duloxetine slightly increased MAP and HR (p<0.05 for MAP) and had no significant effect on respiration rate (table 1, Figure 5). No correlations were found between the mean arterial pressure and URS-EMG values (MAP vs. URS-EMG QP: Pearson coefficient R square = 0.082; MAP vs. URS-EMG CMG: Pearson coefficient R square = 0.006).

**Table 1:** Time course of cardiorespiratory parameters.

**Parameter**	**Saline 0.5h**	**V1 2h**	**V2 2.5h**	**V3 3h**	**V4 3.5h**	**V5 4h**	**V6 4.5h**	**V7 5h**	**Duloxetine 5.5h**
**MAP (mmHg)**	139.58 ± 6.28	134.33 ± 5.56	136.73 ± 6.8	133.50 ± 6.14	131.56 ± 6.69	133.00 ± 6.1	131.08 ± 6.79	129.53 ± 5.47	147.96 ± 6.96*
**HR (beats/min)**	209.93 ± 8.97	203.25 ± 6.07	204.44 ± 7.37	208.12 ± 6.97	208.13 ± 7.48	209.36 ± 7.24	206.51 ± 7.06	210.01 ± 7.21	213.75 ± 11.99
**RR (cycles/min)**	15.02 ± 0.57	14.64 ± 0.60	14.45 ± 0.62	14.35 ± 0.67	14.44 ± 0.64	14.58 ± 0.66	14.58 ± 0.65	14.79 ± 0.66	15.23 ± 0.60

Values show mean arterial pressure (MAP), heart rate (HR) and respiration rate (RR) throughout the experiment. Data were collected during the QP. Data are from 12 cats for MAP and HR and from 11 cats for RR; data from one cat were excluded because the respiration signal was noisy and impossible to be accurately quantified. Statistical significance was tested using ANOVA (Friedman nonparametric test) followed by Dunn’s posthoc test. Asterisk (*) indicates significant changes (p<0.05), relative to V7 and also to saline.

### Histology

**Figure 6:**
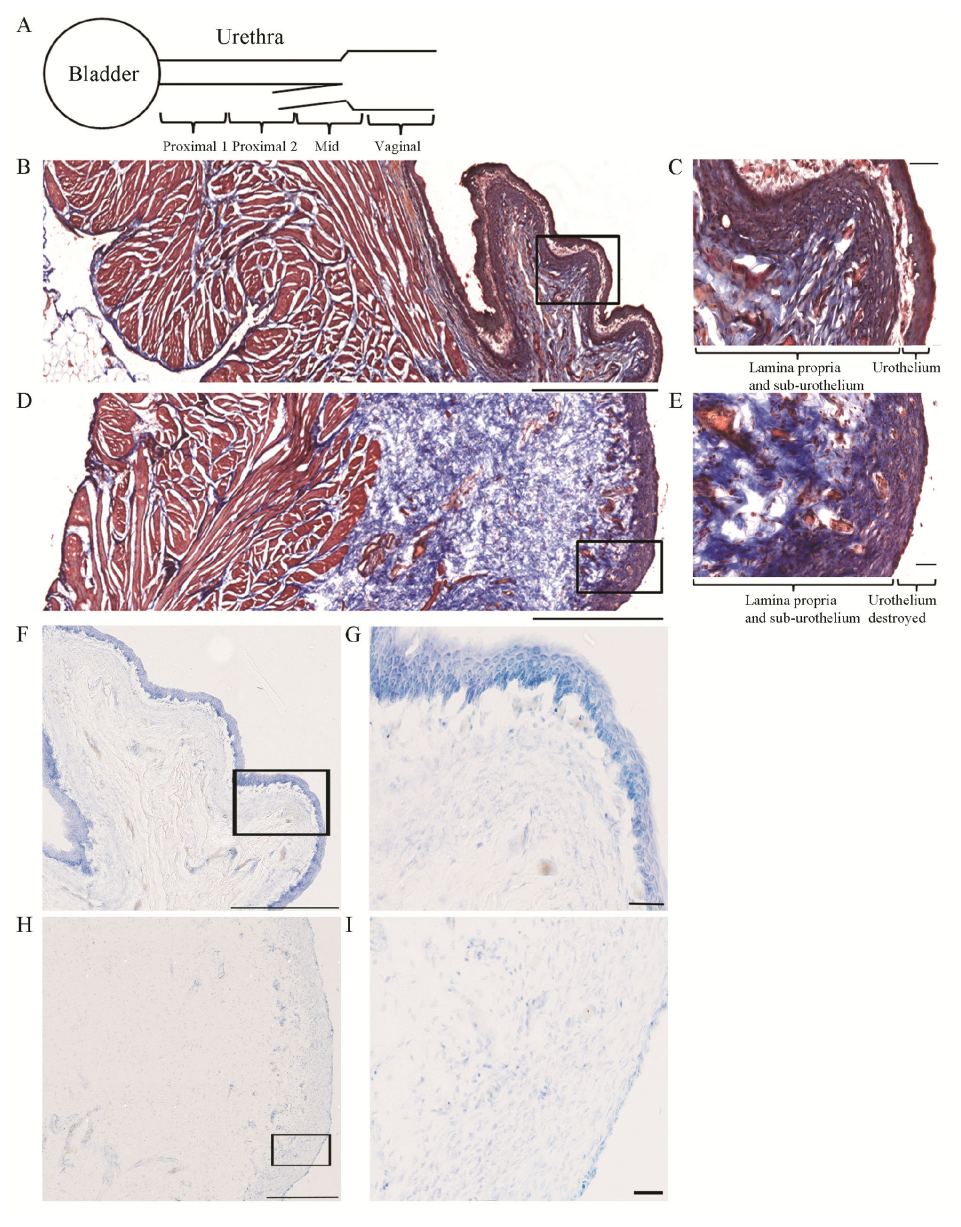
Histological evaluation of bladder tissue. A. Schematic of the preparation of bladder and urethra tissue for cryostat blocking and cutting. Bladder was cut in the longitudinal orientation. Urethra was cut in 4 blocks of 10 mm each, indicated by Proximal 1, Proximal 2, Mid and Vaginal, and transverse sections were cut from each block. B-E. Masson’s trichrome examples from a saline (B, C) and 0.5% acetic acid (D, E) infused bladder. Muscle tissue stains red/pink, collagen fibers stain blue, and nuclei stain black. B. 10x montage image of saline infused bladder tissue. Black box is enlarged in C. C. 40x image illustrating intact urothelium, sub-urothelium and lamina propria. D. 10x image montage of 0.5% acetic acid infused bladder tissue. Black box is enlarged in E. E. 40x image illustrating the lack of urothelium and edema in the sub-urothelium and lamina propria areas. F–G. Toluidine blue examples from a saline (F,G) and 0.5% acetic acid (H,I) infused bladder. F. 10x image montage of saline infused bladder tissue. Black box is enlarged in G. G. 40x image illustrating intact urothelium, sub-urothelium and lamina propria. H. 10x image montage of 0.5% acetic acid infused bladder tissue. Black box is enlarged in I. I. 40x image illustrating the lack of urothelium, edema and infiltration of presumed neutrophils and other cell types in the sub-urothelium and lamina propria areas. Scale bars are 1000 µm for B and D, 500 µm for F, H and 50 µm for C, E, G, I. Images in F, H are from adjacent sections of B, D respectively. Data included are from 2 cats with bladder irritation and 2 controls.

**Figure 7:**
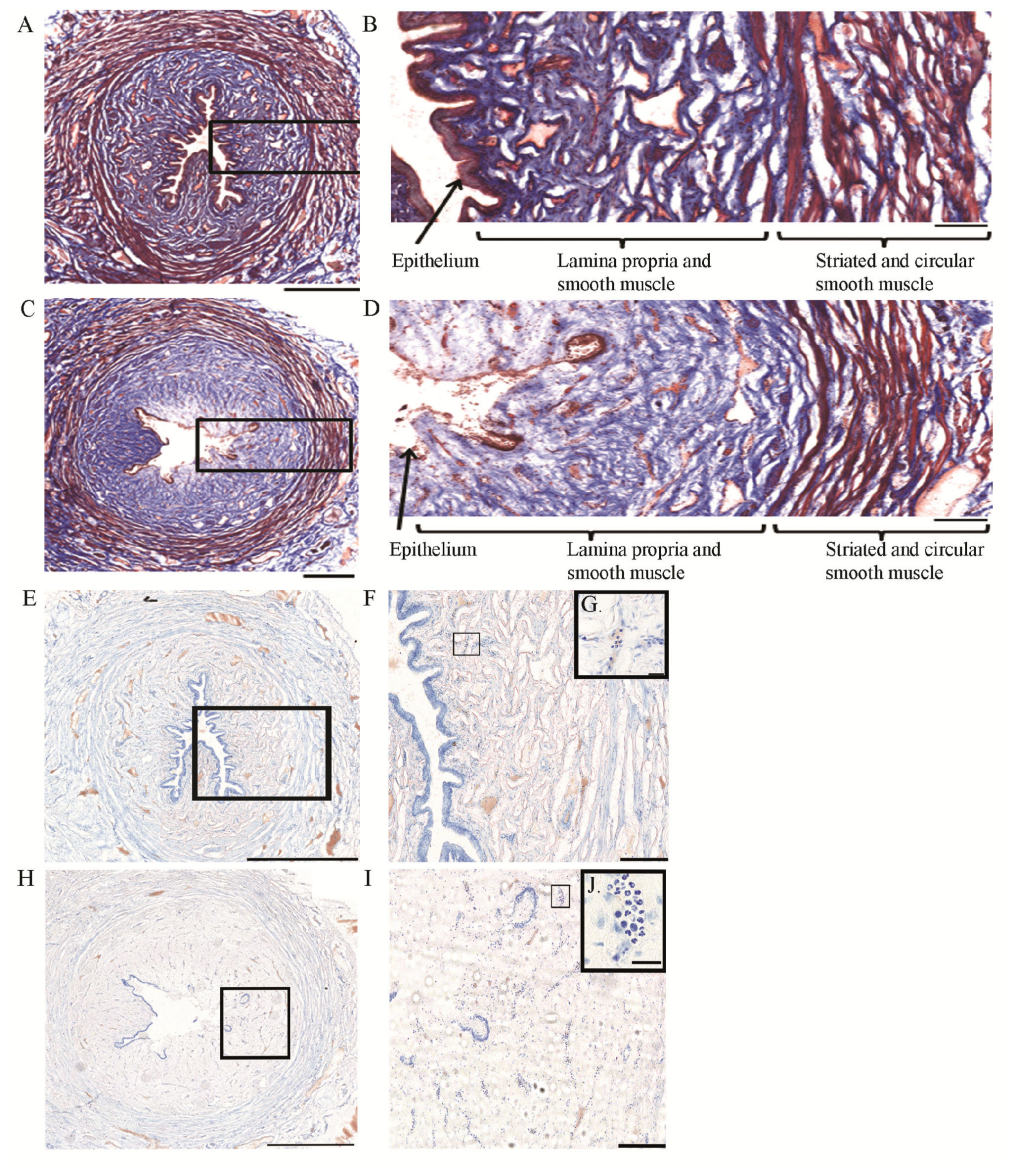
Histological evaluation of urethral tissue. A-D. Masson’s trichrome examples from a saline (A, B) and 0.5% acetic acid (C, D) infused urethra. Sections shown are from mid part of the urethra. A. 10x image montage of saline infused urethral tissue. Black box is enlarged in B. B. 10x image illustrating intact epithelium, lamina propria/smooth muscle. C. 10x image montage of 0.5% acetic acid infused bladder tissue. Black box is enlarged in D. D. 10x image illustrating the lack of epithelium and edema in the lamina propria/smooth muscle area. E–J. Toluidine blue image montage examples from a saline (E–G) and 0.5% acetic acid (H–J) infused urethra. E. 10x image montage of saline infused urethral tissue. Black box is enlarged in F. F. 10x image illustrating intact epithelium, lamina propria/smooth muscle. G. 60x enlargement illustrating a presumed group of neutrophils. H. 10x image of 0.5% acetic acid infused bladder tissue. Black box is enlarged in I. I. 40x image illustrating the lack of epithelium, edema and infiltration of presumed neutrophils and other cell types in lamina propria/smooth muscle area. Note the increased number of presumed neutrophils. J. 60x enlargement illustrating a presumed group of neutrophils. Scale bars are 1000 µm for E, H, 500 µm for A, C, 200 µm for B, D, F, I and 20 µm for G, J Images in E, H are from adjacent sections of A, C respectively. Data included are from 2 cats with bladder irritation and 2 controls.

Masson’s trichrome staining revealed two main differences between saline and acetic acid infused preparations, which were consistent in both bladder (Figure 6) and urethra (Figure 7). First, in the acetic acid irritated preparations, the urothelium in the bladder and the epithelium along the urethra were greatly damaged. Although not quantified, in the bladder, the urothelium was detached from the lamina propria in >90% of the luminal surface, while in the urethra the epithelium was damaged or detached from the lamina propria in > 50% of the luminal surface. No signs of epithelial damage were seen in the saline infused preparations. Second, in the acetic acid irritated preparations, the lamina propria appeared more diffuse and swollen, up to three times larger than in control preparations. The detrusor and urethral smooth muscle, and urethral rhabdosphincter muscle, appeared similar in both groups. Toluidine blue staining revealed increased number (up to ~5x, not quantified) of presumed neutrophils in the acetic acid irritated preparations, particularly noticeable in the urethra (Figure 7). Neutrophil accumulation in the irritated bladder appeared minimal (Figure 6). There was no noticeable neutrophil accumulation in the detrusor and urethral smooth muscle or urethral rhabdosphincter muscle.

## Discussion

### Stability of URS-EMG activity

Under alpha-chloralose anesthesia with saline infusion of the bladder, URS-EMG activity was minimal but when present, it was well correlated with bladder activity, with elevated levels during filling and cessation when bladder contracted. Acetic acid infusion into the bladder increased the URS-EMG levels, expressed as either RMS or spikes/s (figures 2, 4), similar to previous reports [10,12]. Throughout the experiment, URS-EMG activity was higher, almost double, during QP than during CMG (compare Figure 4A with 4C or 4E with 4G), with increasing and decreasing trends with experimental manipulations being similar. This indicates that reliable data could be obtained by introducing QP in the protocol. In addition, this period may be more appropriate for evaluating effects of drugs that decrease the URS-EMG activity. After one hour of acetic acid irritation the URS-EMG activity was not sufficiently stable for drug testing, however, after the 2^nd^ hour the URS-EMG activity levels became stable, varying less than 25% (Figure 4). This suggests that effects of compounds that produce changes on this parameter smaller than 25% of control may not reliably be detected in this model. The time course of the irritation is consistent with previous reports which have shown an early increase in URS-EMG activity due to urethral irritation followed by a late apparent decrease in URS-EMG activity which may result from extreme bladder irritation [12,46]. The initial increase in activity is considered a spinal urethrourethral reflex as it has been shown that it is elicited when the urethra was exposed to acetic acid but not when only the bladder was exposed and it persisted after acute spinal cord transection [12]. Similar results have been reported in rats [46]. The second phase, when the activity decreases, may be related to extreme irritation of the bladder [12]. As previously reported [10], the positive control drug duloxetine (1mg/kg) greatly increased URS-EMG activity (Figure 4).

### Stability of bladder function

Bladder function was assessed under several experimental conditions: saline intravesical infusion, irritation with intravesical acetic acid and after systemic duloxetine treatment. During saline infusion, all cats showed large rhythmic voiding contractions. Acetic acid irritation for at least 1h reduced bladder capacity and bladder contraction amplitude but not to a statistically significant extent, and significantly increased voiding frequency (figures 2, 3), similar to previous reports [10]. Duloxetine reversed the acetic acid induced changes by decreasing voiding frequency and increasing bladder contraction amplitude (Figure 3), similar to previously published results in cat irritated with acetic acid [10] and in women with OAB [47]. In summary, in this acute model of OAB, 1h of acetic acid infusion was sufficient to irritate the bladder and the preparation was stable for more than 5h, allowing reliable drug testing.

Histological evaluation of acetic acid induced bladder and urethra tissue damage and possible correlations with bladder and URS-EMG activity.

Prolonged infusion of acetic acid greatly damaged the urothelium of the bladder and the epithelium of the urethra (figures 6, 7), causing edema and infiltration of neutrophils in the lamina propria, consistent with early phase of inflammation. These results are similar to those from a study in the rat which found similar damage of urothelium and lamina propria and infiltration of neutrophils and/or macrophages 9 hours following only 5 minutes of 0.75% acetic acid infusion in the bladder [48].

Exposure to acetic acid (low pH) in the bladder and urethra can activate ASIC receptors located in epithelial cells which in turn can cause release of ATP and activation of P2X2/3 receptors in the primary afferent neurons [49–51]. As the epithelial layer was damaged, acetic acid presumably reached nerve fibers including sensory fibers positive for Calcitonin gene related peptide (CGRP) that terminate in the epithelial layer and lamina propria in the bladder [52] and urethra [16,53]. The noxious stimulation may cause the release of CGRP or Substance P (SP) from nerve terminals, which may further stimulate epithelial cells to release transmitters that can modulate afferent nerves function [54]. It also has been shown that noxious stimulation of primary afferent fibers causes release of CGRP and SP in the spinal cord in the cat [55]. Together, this strong stimulation of afferent activity, peripheral and central, may explain increases in bladder contraction frequency and URS-EMG activity (figures 4, 5). In addition, NOS positive fibers reach the subepithelial and epithelial layers in the urethra [53] and damage of these fibers may impair urethra function. Duloxetine likely suppresses bladder frequency through reduction of nociceptive inputs to bladder and facilitation of somatic outflow to sphincter [56].

While the epithelium of bladder and urethra and areas directly underneath them (i.e. lamina propria) were greatly affected by acetic acid, the smooth muscle of the bladder and urethra as well as the striate muscle of the urethra did not appear altered. This information should be considered when testing compounds targeting receptors specifically expressed in each structure.

### Stability of vital signs and blood chemistry

Changes in the cardiovascular and/or respiratory parameters are usually due to changes in the depth of anesthesia and/or experimental manipulations (e.g. drugs). These changes can affect blood acid–base balance inducing acidosis or alkalosis, which in turn can affect bladder/urethra function, increasing data variability and making data interpretation difficult. To achieve stable levels of anesthesia throughout the experiment we recommend placing the animals on a continuous infusion of alpha-chloralose (5mg/kg/h) after the initial bolus of 65-75mg/kg. Under these experimental conditions, in our hands all cardio and respiratory parameters monitored were stable (most of them varying less than 5%), throughout the duration of the vehicle testing, regardless of the bladder treatment (table 1, Figure 5), making this preparation suitable for testing the effects of various drugs not only on LUT but also on cardiac or respiratory systems, with the known caveats of alpha-chloralose anesthesia. Some of these caveats include an actions of alpha-chloralose on various receptors (such as potentiation of GABA_A_ receptor activity [57]), potential for altering cardiovascular responses to adrenergic drugs [58,59], and induction of hypothermia in cats [60]. In our preparations, blood pH values, hemoglobin status and electrolyte composition were stable. While we could not find literature for cats under similar conditions (i.e. LUT irritation under alpha-chloralose anesthesia), the last column in table 2 summarizes parameters from few sources, some measured in awake cats [61] and from Cornell veterinary program (http://ahdc.vet.cornell.edu/sects/clinpath/reference/blood.cfm). The values obtained from our cats are comparable to these, suggesting that the experimental procedures (i.e. anesthesia, LUT irritation) have no significant effect on blood acid–base balance.

**Table 2:** Blood chemistry.

	**Parameter**	**Saline 0.5h**	**V2 2.5 h**	**V5 4.0h**	**Duloxetine 5.5h**	**Reference values from [61] and #**
Acid/Base status	pH	7.32 ± 0.01	7.31 ± 0.01	7.32 ± 0.01	7.32 ± 0.01	7.21-7.41
	PCO_2_ (mmHg)	34.25 ± 0.95	35.58 ± 1.29	34.17 ± 0.92	34.70 ± 1.15	28-50
	HCO_3_ (mmol/L)	16.41 ± 0.40	16.65 ± 0.55	16.43 ± 0.34	16.63 ± 0.35	16-24.1
	AnGap (mmol/L)	26.16 ± 0.67	27.75 ± 1.36	26.36 ± 0.23	26.02 ± 0.38	15-23
	tCO_2_ (mmol/L)	17.46 ± 0.42	17.73 ± 0.59	17.48 ± 0.37	17.69 ± 0.38	17-24
	BE	-8.02 ± 0.39	-6.29 ± 1.58	-7.75 ± 0.27	-7.56 ± 0.24	(-8.5) - (-1.5)
Oxygen/ Hemoglobin	PO_2_ (mmHg)	102 ± 4.24	95.67 ± 4.34	94.67 ± 3.29	90.8 ± 1.44	
	tHb (g/dL)	10.2 ± 0.40	10.71 ± 0.48	11.57 ± 0.38	12.5 ± 0.49	10.9-15.7
	SO_2_ (%)	95.92 ± 0.45	95 ± 1.54	95.75 ± 0.39	95.4 ± 0.37	
Electrolytes	Na^+^ (mmol/L)	163.67 ±0.45	164.33 ± 1.54	162.25 ± 0.39	161.4 ± 0.37	149-157
	K^+^ (mmol/L)	3.01 ± 0.12	3.03 ± 0.13	3.15 ± 0.09	3.18 ± 0.07	3.3-4.6
	Cl^-^ (mmol/L)	123.67 ± 0.58	122.83 ± 0.58	122.42 ± 0.42	122 ± 0.52	113-127

Time course of blood gas analysis throughout the experiment. Data are from 12 cats for saline, V2 and V5 time points and from 10 cats for duloxetine time point. In two experiments the blood gas was not analyzed after duloxetine administration.

# indicates values summarized from Cornell veterinary program analysis on awake cats (http://ahdc.vet.cornell.edu/sects/clinpath/reference/blood.cfm) and published reference [61].

## Conclusions

Preparations are healthy and stable for 6-7 h tested. Blood gas analysis, MAP, HR and respiration parameters are constant throughout the experiment, independent of the manipulations that irritate the bladder and/or increase the URS-EMG activity. The consistency of this preparation is likely aided by the constant alpha-chloralose infusion for anesthesia maintenance, as opposed to boluses. For assessing drug effects on bladder function, 1-2h of acetic acid irritation is sufficient to induce significant and stable changes. For assessing drug effects on rhabdosphincter activity, a 2h period of acetic acid irritation is recommended, followed by at least one vehicle. The protocol modification with introduction of a quiet period provided more robust data, although the increasing or decreasing trends of URS-EMG activity were similar during QP and CMG. Histological data, indicating great damage to the bladder and urethra epithelium with no apparent damage to the muscle, should be considered when testing targets specific to muscle, nerves or other lower urinary tract structures.

## Supporting Information

Figure S1 **Effects of bolus alpha-chloralose on blood pressure, bladder pressure and URS-EMG activity.** Note strong inhibition of URS-EMG activity with minor effects on other parameters. * indicates that a blood sample was taken. & indicates resetting the balance collecting the voided volume.
(TIF)
