# Supplementary figures and images for: Stability of the Acetic Acid-Induced Bladder Irritation Model in Alpha Chloralose-Anesthetized Female Cats
Source: PLoS One. 2013 Sep 9;8(9):e73771. doi: 10.1371/journal.pone.0073771 (PMC3767621; doi:10.1371/journal.pone.0073771)

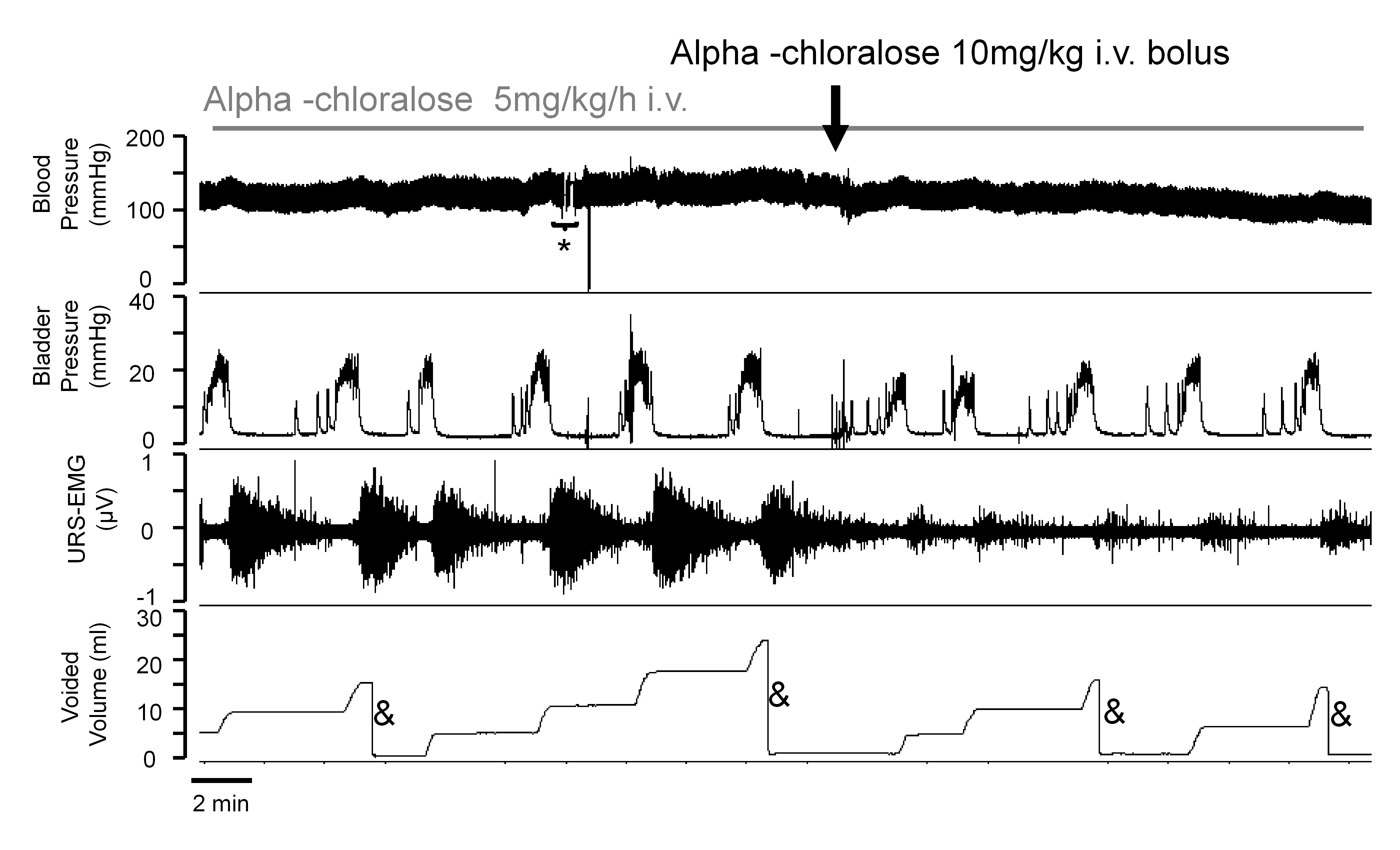

Supplement: Figure S1 — Effects of bolus alpha-chloralose on blood pressure, bladder pressure and URS-EMG activity. Note strong inhibition of URS-EMG activity with minor effects on other parameters. * indicates that a blood sample was taken. & indicates resetting the balance collecting the voided volume. (TIF) [file pone.0073771.s001.tif]
